# Sustained release from a metal - Analgesics entrapped within biocidal silver

**DOI:** 10.1038/s41598-017-03195-w

**Published:** 2017-06-23

**Authors:** Barak Menagen, Rami Pedahzur, David Avnir

**Affiliations:** 1 Institute of Chemistry and the Center for Nanoscience and Nanotechnology, the Hebrew University of Jerusalem, Jerusalem, 9190402 Israel; 2 Department of Environmental Health, Hadassah Academic College, Jerusalem, 91010 Israel

## Abstract

Matrices for sustained release of drugs have been based on polymers, biomaterials and oxides. The use of the major family of metals as matrices for sustained release is, to the best of our knowledge, unknown. In this context we describe a new family of bio-composites for sustained release of drugs, namely analgesic drugs entrapped within metallic silver. Synthetic methodologies were developed for the preparation of ibuprofen@Ag, naproxen@Ag, tramadol@Ag and bupivacaine@Ag composites. Detailed kinetic analysis of the release of the drugs from within the metal, is provided, demonstrating that metals can indeed serve as reservoirs for drug release. The metal in our case acts not only as a drug releasing source, but also as an antibacterial agent and this property of the composites was studied. Unexpectedly, it was found that the entrapment of the analgesics within silver, dramatically enhances the growth inhibition activity of wild type *Pseudomonas aeruginosa*, exceeding by far the inhibition activity of the separate components. A mechanism for this interesting observation is provided. The strong antimicrobial activity combined with the analgesic activity open the road for future applications of these materials as dual-purpose components in wound treatment.

## Introduction

**Figure 1:**
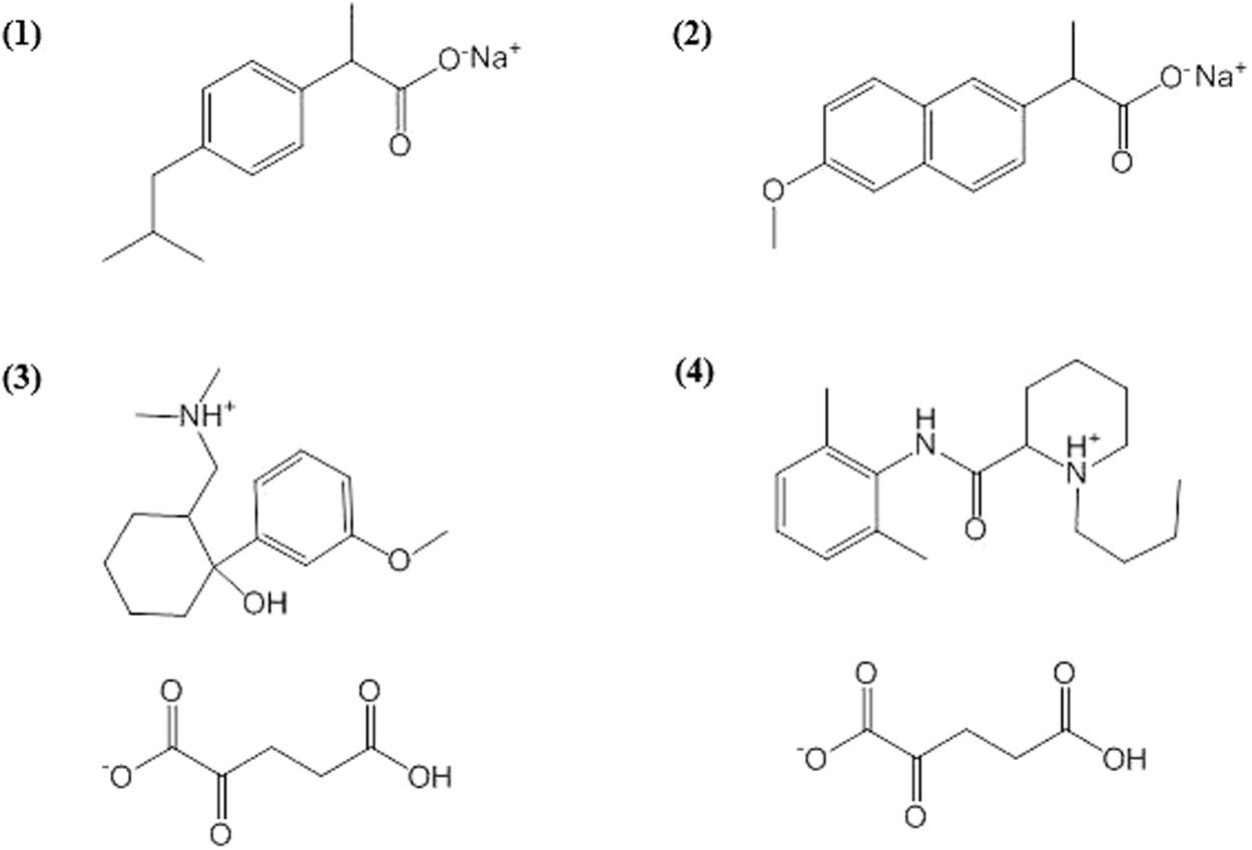
The structures of the analgesics used for entrapment within silver: (1) Sodium ibuprofen. (2) Sodium naproxen. (3) Tramadol HCl, converted to the α-ketoglutarate (shown). (4) Bupivacaine HCl, converted to the α-ketoglutarate. All four drugs are racemates. In their entrapped form, they are the non-ionized carboxylic acids and free amines.

Sustained release of drugs is a major, well developed field of biomaterials; hundreds of drug-holding matrices are known^1–4^. The main families of materials – organic materials (such as polymers) and inorganic materials (such as SiO_2_, ZnO and TiO_2_) have been intensively researched and developed for this application. It comes as a surprise that the third major family of materials – elemental metals – has not been used as a 3D drug releasing matrix. As sustained release of drugs has many important merits - improving the effectiveness, reducing toxicity, improving patient compliance, and more, opening the road to an additional major class of materials, is of interest^5, 6^. Here we describe the first successful steps in that direction, along with an unexpected benefit: enhancement of bioactivity of the organically doped metal. Specifically we describe the entrapment of the analgesic drugs ibuprofen (NSAID), naproxen (NSAID), tramadol (opioid), and bupivacaine (anesthetic) – which represent three major analgesics families, within metallic silver﻿ (Fig. 1). These drugs are well known and widely used for topical treatment and appear also in sustained release formulations^7–16^.

We report the successful release of these drugs from the corresponding drug@silver composites; we describe and analyze in detail the release kinetics; and we describe methods for controlling the release profiles. Silver was selected because of its potential dual purpose: A releasing matrix, and an agent of known antibacterial properties^17^. The effects of the analgesic dopants on this biological activity have been studied, and, unexpectedly, it was found that the bacterial growth inhibition of the analgesic-loaded silver, by far exceeds the activity of the separate components. A mechanism for this enhanced activity, which relies on the increased release rate of Ag^+^ mediated by the entrapped drug molecules, is proposed. Analgesic drugs were selected for exploration of the idea of using metals as release-reservoirs because of a potential longer term application in wound treatment, were solutions for sustained release infection control which go hand-in-hand with wound pain management, are in need (see Outlook).

What has enabled the developments described in this report is the advent of a new, powerful materials methodology, which enables the doping of metals with molecules, polymers and enzymes^18–21^. Many applications, all across chemistry, have been demonstrated with these new composites, and these are summarized in a recent review^22^. We were motivated to explore the potential use of these new materials for sustained drug release by a specific observation, namely that the biocidal activity of either silver or copper doped with (antibacterial) chlorhexidine was order of magnitudes higher than that of the separate components; synergistic action of both chlorhexidine and metal cations was proposed as a mechanism for the enhanced activity^23–25^.

Basically, the approach for obtaining molecule@metal, involves the reduction of the metal cation in the presence of the molecules to be entrapped^18–22^. The apparent simplicity of this approach by no means hints to synthetic simplicity. On the contrary, many parameters have to be controlled: Reducing conditions that will not affect the dopant molecules, manipulating the desired dopant so that entrapment ﻿will be preffered﻿ rather than remaining in solution, easy discarding of the synthetic reganets and in their products, formation of a metallic matrix that, on the one hand will tightly hold the dopant molecules and on the other will allow their release; formation of adequate metal porosity for the release, and so on. The results of this optimisation parameters search are described in the Experimental Details section.

## Experimental Details

### Chemicals and bacterium

Silver nitrate, sodium hypophosphite hydrate, tramadol hydrochloride, bupivacaine hydrochloride monohydrate, sodium ibuprofen salt and Muller Hinton broth (MHB) were purchased from Sigma Aldrich. Ibuprofen acid and sodium naproxen were purchased from Acros Organics. α-ketoglutaric acid was purchased from Chem-Impex International. Wild type *Pseudomonas aeruginosa* (PAO1) was kindly obtained from Prof. E. Banin at Bar-Ilan University.

### Synthesis of NSAIDs@silver: Ibuprofen@Ag and naproxen@Ag

The NSAID@silver composites were prepared as follows: 0.211 g (9.24 × 10^−4^ moles) of ibuprofen sodium or 0.233 g (9.24 × 10^−4^ moles) of naproxen sodium was added to a 10 mL double-distilled water (DDW) solution of 1.57 g (9.24 × 10^−3^ moles) of silver nitrate. The solution was kept at 15 °C and stirred at 500 rounds per minute (RPM). After 5 minutes, 0.6 g (6.82 × 10^−2^ moles) of sodium hypophosphite was added and the entrapment process was let to proceed for 24 hours. The resulting suspension was filtered, washed with 10 mL of DDW and vacuum-dried overnight. After 1 day of storage the composite was washed again with four portions of 10 mL of DDW and vacuum-dried for 5 days. The resulting powder was crushed and homogenized. The yields were 0.91 g of ibuprofen@Ag (ibu@Ag) and 1.02 g of naproxen@Ag (nap@Ag), both in the non-ionized form of the carboxylic acids. For comparative purposes, pure silver (0.87 g) was prepared by the same method but without adding any analgesics. It should be noted that the amount used reflect standardly used quantities: For instance, regarding silver, up to10 mg/cm^3^ of silver on wound dressing is used^26^; regarding ibuprofen, 0.5 mg/cm^3^ of ibuprofen is used in wound dressing preparations^9^.

### Synthesis of tramadol@Ag and bupivacaine@Ag

For efficient entrapment these two drug molecules, the counter anion – chloride – must be replaced. The anion exchange into α-ketoglutarate was carried out as follows: For tramadol, 0.278 g (9.24 × 10^−4^ moles) of hydrochloride salt was added to 5 mL of 1 M of sodium hydroxide solution within a centrifuge tube, and the suspension mixed for 5 minutes. The tube was then centrifuged for five minutes at 5,000 RPM. The supernatant was discarded and 1.0 mL of DDW was added along with 0.135 g of alpha-ketoglutaric acid (9.24 × 10^−4^ moles). For bupivacaine 0.6 g of the hydrochloride monohydrate salt was added to 10 mL of 1 M sodium hydroxide and mixed for five minutes. The suspension was filtered and vacuumed overnight. Then, 0.267 g (9.24 × 10^−4^ moles) of bupivacaine was added to 1 mL DDW containing 0.135 g (9.24 × 10^−4^ moles) of α-ketoglutaric acid. The second step is the entrapment process: 1.57 g of silver nitrate was dissolved with 9 mL of DDW. The vial was kept at 15 °C for five minutes, while stirring at 500 RPM. Then 0.6 g of sodium hypophosphite monohydrate was added and after 30 seconds the 1.0 mL containing 9.24 × 10^−4^ moles of tramadol α-ketoglutarate or bupivacaine α-ketoglutarate was added. The entrapment process took 24 hours. The resulting composite was filtered and washed with 10 mL of 1 M sodium hydroxide in order to convert the drug salt into the free amine form. The composites were vacuum-dried overnight, then washed with additional 10 mL of sodium hydroxide and with 30 mL DDW. Finally, the product was vacuum-dried for five days, and the powder crushed and mixed. The yields of tramadol@Ag)tra@Ag(and bupivacaine@Ag (bup@Ag) were 1.16 g and 1.05 g, respectively. 0.87 g of undoped silver was prepared similarly for comparative purposes, without adding any analgesics. All four purified biomaterials were found to be composed of only two components: The metal and the entrapped drug – careful chemical analytical checks revealed no other residues.

### Sustained release measurements

These were carried out in DDW and were followed by UV spectroscopy, as detailed in Table 1. Initial concentrations (Table 1) were tailored for optimal detection. The suspensions were stirred at 300 RPM at room temperature. At specific times, a sample of 3 ml from the suspension was transferred to a quartz cuvette and the amount of the released drug measured at the maximum wavelength of the UV adsorption spectrum. Then, the sample was immediately transferred back to the tested suspension. When needed, centrifugation prior to measurement was carried out (5 seconds at 3000 RPM). Release of the NSAIDs was also measured on disks, which were prepared by compressing 200 mg of the composite powder at 9,000 PSI for 5 min by using an infra-red (IR) pellets press that formed 13 mm diameter disks^27^. The release measurements from the disks were carried out as for the powders.

**Table 1:** Some details of the sustained release measurements.

	Ibu@Ag	Nap@Ag	Tra@Ag	Bup@Ag
Wavelength (nm)	222.0	230.0	270.5	263.0
Concentration (mg/mL)	0.2	0.02	1	4
Total volume (mL)	50	500	25	25

### Measurements of the silver ion concentrations

Silver ion concentrations were measured by inductively coupled plasma mass spectrometry (ICP-MS) as follows: The tested material was dispersed in 3.0 mL of MHB at 37 °C within an incubator shaker at 150 RPM. After four hours, a sample of 50 microliter was taken from the tested suspension and diluted into an Eppendorf tube containing 450 microliter of DDW. Then the sample was centrifuged at 5,000 RPM for five minutes. Afterwards, 250 microliter of the centrifuged sample was diluted with 2.25 ml of nitric acid, 1 M. The acidic sample was taken to the ICP-MS measurement.

### Minimum inhibition concentration (MIC) tests

*Pseudomonas aeruginosa* (PAO1) was kept on nutrient agar plates in 4 °C. Single colonies were taken and mixed in 25 mL of MHB and incubated for 18 hrs in an incubator shaker at 37 °C at 150 RPM. Then a sample of 0.5 mL of the overnight (O.N.) culture was diluted in 50 mL fresh MHB. After 90 min, 3.0 mL were transferred to a sterile plastic cuvette and the optical density (O.D) at 600 nm was determined. Afterwards, the active agents were added to the cuvette which were incubated at 37 °C in the incubator shaker at 150 RPM and the O.D was followed every 30 min for 4 hrs. The first step was to determine the minimum inhibitory concentration (which is the minimum concertation of a substance that is needed to inhibit the growth of a bacterial culture under a specific set of experimental conditions as visualized by optical measurements) for all composites. Then, the activity of the composites has been compared with that of its individual compounds, either alone or in simple mixtures thereof according to their molar ratio in the composite. As the NSAIDs@Ag and the amines@Ag cause slight pH variations (pH of 6.40 and 7.55 respectively), the possible effect of these pH changes was checked and compared to the standard control which has a pH of 7.0. The acidic pH was checked by using the same molar ratio of the entrapped ibuprofen in solution. The basic pH could not be tested with tramadol free amine, and therefore it was checked by adjusting the pH with sodium hydroxide.

### Instrumentation

Thermogravimetric analysis (TGA) was measured with a Mettler-Toledo TGA/SDTA 851e, from 50 to 800 °C, at a heating rate of 10 °C per minute under air atmosphere. Density measurements were carried out with a Micromeritics AccuPyc 1340 instrument using helium as the displacing gas. UV – Vis absorbance spectroscopy was carried out with Hewlett-Packard 8452 A diode-array UV-vis spectrophotometer. ICP-MS measurements of Ag^+^ were carried out with Agilent 7500 cx. SEM (scanning electron microscope) and EDS (energy-dispersive X-ray spectroscopy analysis) were carried out on a Sirion (FEI) high resulotion (HR) SEM instrument. X-ray powder diffraction measurements were performed on a D8 Advance diffractometer (Bruker AXS, Karlsruhe, Germany) with secondary Graphite monochromator, 2° Sollers slits and 0.2 mm receiving slit. The powder samples were placed on low background quartz sample holders. XRD (X-ray diffraction) patterns from 5° to 85° 2θ were recorded at room temperature using CuKα radiation (λ = 0.15418 nm) with the following measurement conditions: tube voltage of 40 kV, tube current of 40 mA, step scan mode with a step size of 0.02° 2θ and a counting time of 1 s per step. The value of crystallite size was determined from the experimental XRD data using Scherrer equation. The instrumental broadening was determined using LaB6 powder (NIST SRM 660).

## Results and Discussion

### The Biocomposites

#### Formation

The four drugs represented different challenges for their entrapment, as two of them – the NSAIDs ibuprofen (ibu) and naproxen (nap) - are aromatic carboxylic salt compounds, while the opioid tramadol (tra) and the anesthetic bupivacaine (bup) are quaternary ammonium compounds (Fig. 1). The entrapment process involves the reduction of silver nitrate with hypophosphite according to (equation 1):

1 $$2{{\rm{AgNO}}}_{3}+{{\rm{NaH}}}_{2}{{\rm{PO}}}_{2}+{{\rm{H}}}_{2}{\rm{O}}\to 2{\rm{Ag}}+{{\rm{NaH}}}_{2}{{\rm{PO}}}_{3}+2{{\rm{HNO}}}_{3}$$

It is seen that the reaction mixture is acidified as a result of the reduction. This drop in pH enhances the entrapment of the NSAIDs because the water soluble sodium salts are converted to the non-ionized acids, which are much less water-soluble. As a consequence, the drug molecules are not readily washed out from the interior of the metallic matrix, making this outcome suitable for sustained release. The two amine drugs, would have remained in their water soluble HCl ammonium form, and therefore represented a different challenge for entrapment. The methodology developed here – which we believe is relevant to similar cases as well – was to replace the chloride with another anion that would significantly decrease the water-solubility of the drug and has functional moieties that will interact strongly with the metallic crystallite surface. α-ketoglutarate, a dicarboxylic compound (Fig. 1) was found to be most suitable for that purpose. Then, after entrapment, that linker α-ketoglutarate, is extracted by washing the composite with sodium hydroxide, resulting in the non-ionized free amine entrapped within the metal, again a desirable merit for sustained release applications. In section 3 below, we show that these synthetic strategies successfully addressed the release requirements, tailored from minutes, hours to days.

### Characterization

#### ibu@Ag and nap@Ag

**Figure 2:**
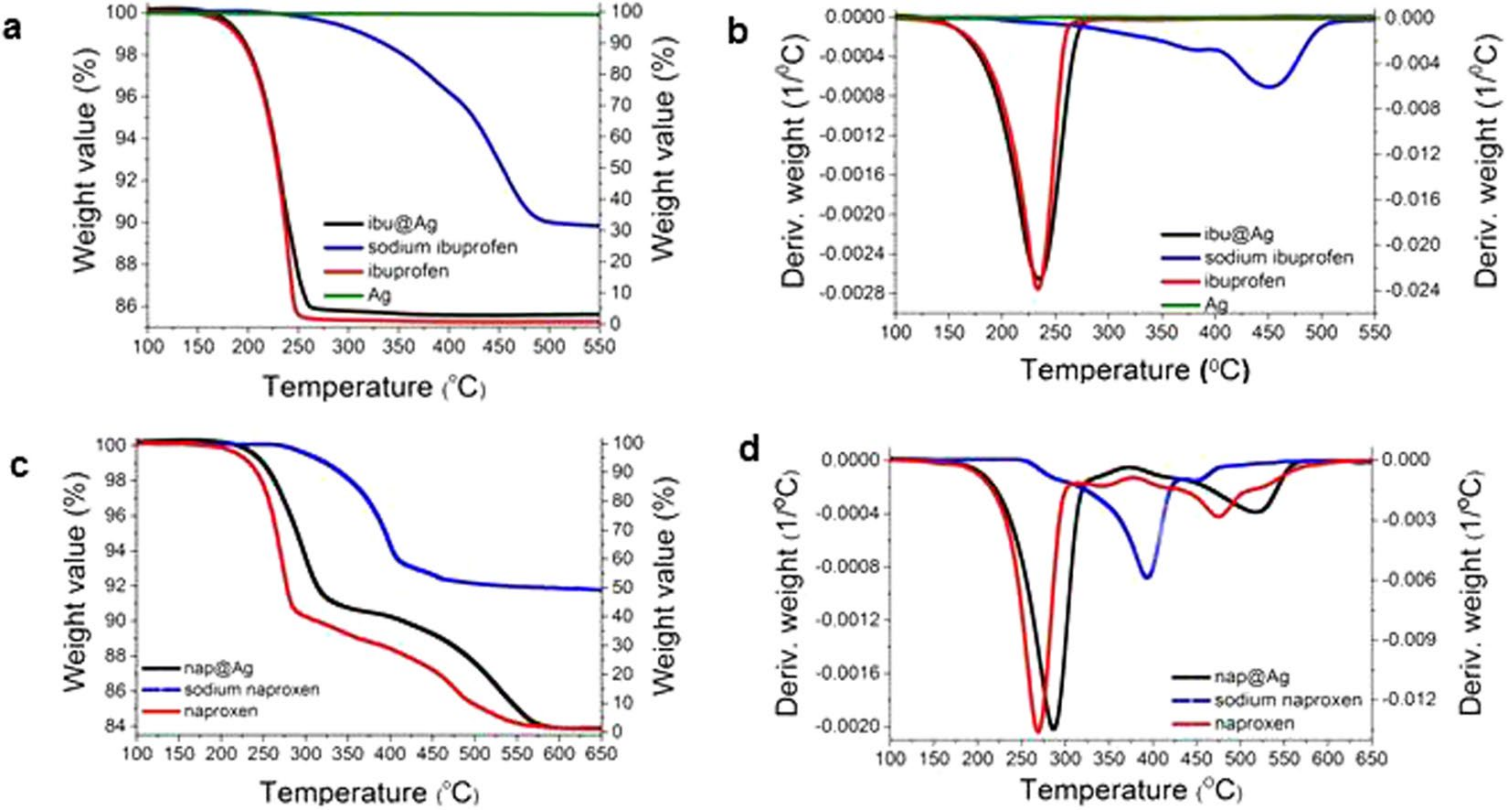
The weight loss vs. temperature (TGA) and the weight loss first derivative graphs of the composites and of the pure components. (**a**) and (**b**): ibu@Ag (black line, left axis) and Ag (green line, left axis), sodium ibuprofen (in blue, right axis), ibuprofen (in red, right axis). (**c**) and (**d**): nap@Ag (in black, left Y axis), sodium ibuprofen (in blue, right axis), ibuprofen (in red, right axis).

**Figure 3:**
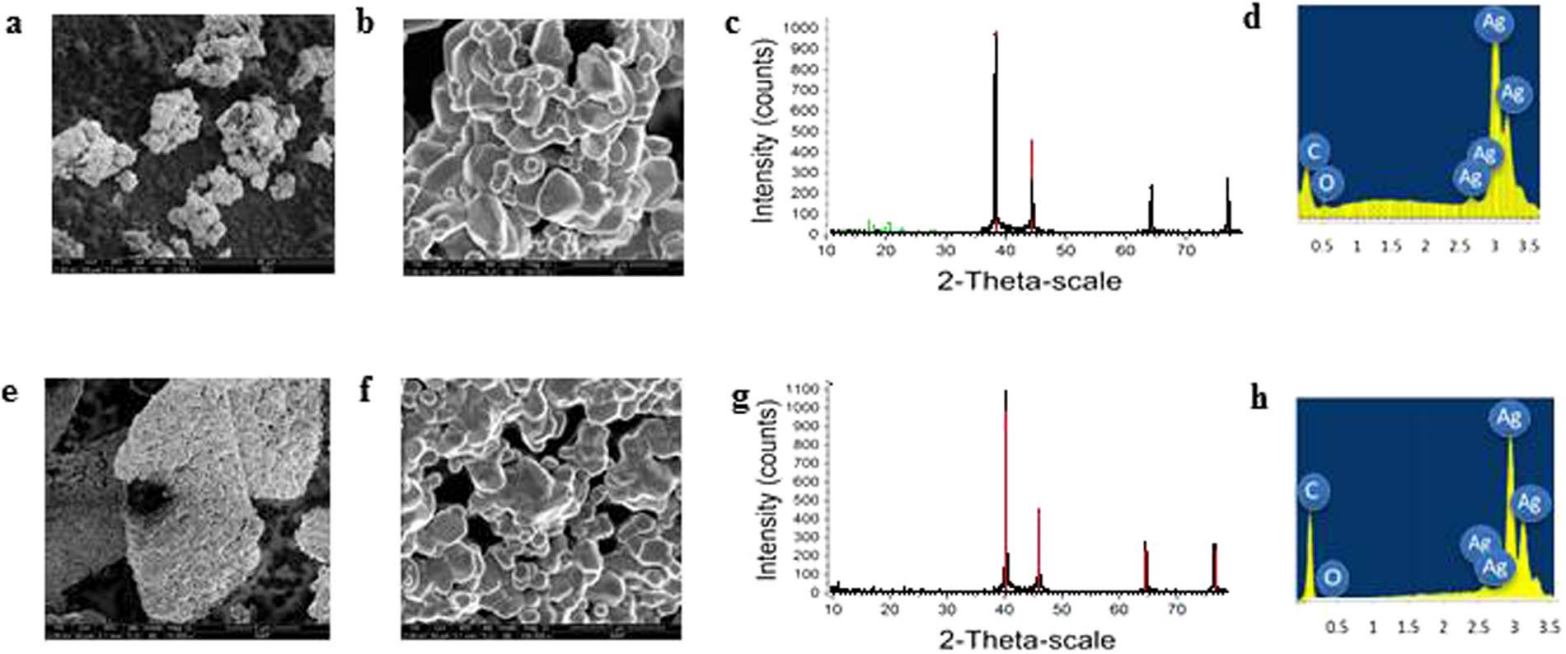
(**a**) – (**d**): ibu@Ag. SE-SEM: (**a**) bar - 40 µm, (**b**) bar - 2 µm. (**c**) XRD: black - ibu@Ag XRD; red - literature silver^58^; green - literature ibuprofen^58^ (**d**) EDAX. (**e**) – (**h**): nap@Ag. SE-SEM: (**e**) bar - 5 µm, (**f**) bar - 1 µm. (**g**) XRD: black - nap@Ag; red pure silver (**h**) EDAX:

The TGA of ibu@Ag (Fig. 2a) indicates a successful entrapment of ibuprofen within silver at a level of 14.5% by weight (69.2% entrapment yield) – both the free drug and the entrapped one decompose at the same temperature of 234 °C (Fig. 2b). As emphasized in the Experimental Details, it was important to entrap all four drugs in their native non-ionized form, which contributes to the ability to sustain the release of the drugs. The TGA indeed proves that the ibuprofen acid and not the sodium salt, was entrapped (the residue above 500 °C most probably belongs to inorganics residual). The TGA of nap@Ag (Fig. 2c), indicates an entrapment of 16.1% (77.2% entrapment yield) and has general features similar to ibu@Ag: The thermal decompositions of free naproxen and of the entrapped drug are quite similar, while the sodium salt needs higher temperatures to decompose (Fig. 2d). On the microscopic level, one sees a hierarchical structure, starting – Fig. 3a,e – as a collection of aggregates several tens of µm in size. This granular material is then composed of sub-micron aggregated doped silver particle (Fig. 3b and f), which, in turn, are composed of elementary silver nanocrystals 40–50 nm in size (ibu@Ag: 46 nm, nap@Ag: 40 nm). The sizes of these elementary particles were obtained from the XRD spectra (Fig. 3c and g) by using Scherrer’s equation; XRD also confirm the pure silver nature of these nanocrystals. EDAX analyses – Fig. 3d,h – show the hybrid nature of the composites – carbon and oxygen peaks – not seen in the spectrum of pure Ag prepared by the same method (see Figure S1, Supplementary material) – appear along with the typical peaks of silver. The nanocrystals sizes are 115 nm, which indicate that the entrapped molecules interfere with the crystal growth process. Density measurements indicate that the doping of the metal with the organic components significantly reduces the density: While the density of silver prepared without the dopant is 10.34 g/cm^3^, (literature value: 10.49)^28, 29^, the densities of both composites are less than a half - 4.63 and 4.65 g/cm^3^ for ibu@Ag and nap@Ag, respectively.

#### tra@Ag and bup@Ag

The preparation of these composites required a new addition to the existing arsenal of methods for preparing dopant@metal, which did not work for the entrapment of the HCl salts of the two amine drugs. The water solubility of these drugs did not allow the standard mechanism of entrapment to operate. That mechanism (described in detail in previous publications) requires that the adsorptive residence time of the molecule to be entrapped on the surface of the growing metal crystallites, will be long enough to allow their entrapment in this state by further reductive precipitation of the metal^21, 22, 30^. However, the good water solubility of the two drugs, favored dissolution over adsorption. To overcome this we changed, as described above, the counter anion to an anion that will diminish that solubility, namely α-ketoglutarate; we note that this acid is not toxic, as it is part of the Krebs cycle.

Microscopy, EDAX, XRD and TGA data of the amine-drugs@Ag are all presented in Figures S2, S3, S4 and S5 in the Supplementary material, showing the aggregated architectures and the composite nature of these composites (some residual AgCl is detected in the case of bup@Ag - Figure S3). The TGA of tra@Ag – Figure S4a - indicates a loading of 13% (62% entrapment yield). Interestingly, unlike the entrapped acids, the decomposition profiles show similarity of the free HCl salt and the entrapped drug, while the free amine decomposes at almost 100 °C lower. That similarity is a direct indication that the lone-pair electrons of the amine are associated strongly in the interaction with the metallic silver. The TGA of bup@Ag (Figure S5a) indicates a loading of 12% (45% entrapment yield), and in this case all three decomposition profiles - the free drug, its HCl salt, and the entrapped free drug, are quite similar. We propose that the heavy steric hindrance on the nitrogen (compared to tramadol) lowers the effect of the nitrogen interaction on the TGA profile. The densities of tra@Ag and bup@Ag are about 0.5 g/cm^3^ higher compared to the NSAIDs@Ag densities: 5.11 and 5.01 g/cm^3^, respectfully. This is a reflection of the larger elementary crystallite sizes of tra@Ag and bup@Ag compared to the NSAIDs@Ag, namely 80.0 and 61.1 nm, respectively, as determined from the XRD spectra (see Figures S2 and S3, Supplementary material). All of these changes between the two pairs of drugs reflect the changes in the adsorptive molecular moieties (carboxylates vs. amines), the use of the linker in the latter (the ketoglutarate), and the difference in procedures, tailored for these two classes of dopants. As we shall see next, these differences are reflected in the release profiles, and in the bioactivity.

### Sustained release profiles

**Figure 4:**
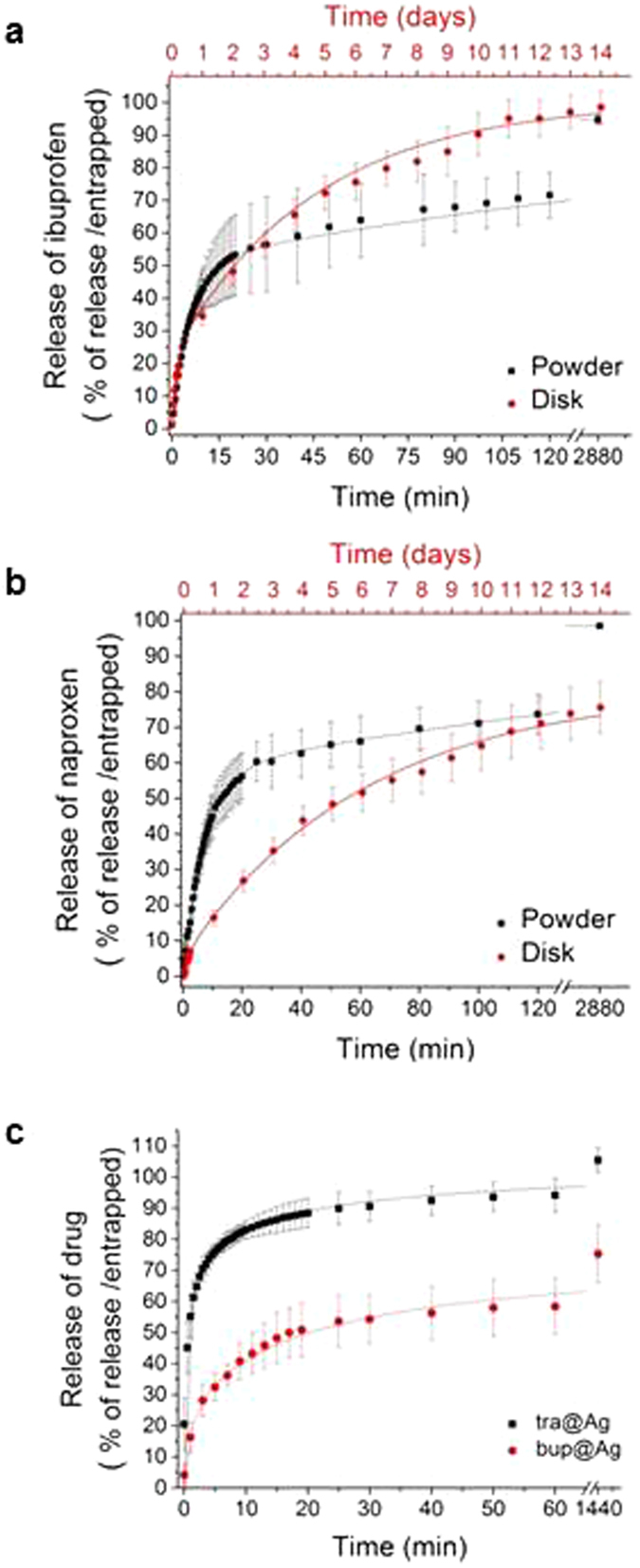
Drug release profiles of (**a**) ibuprofen and (**b**) naproxen from their metallic silver matrices. Black squares: Powder, Red circles: Compressed discs (note the different time scales). Full lines: Fits to the kinetic model of eq. (1) (Table 3; range of fit: 120 min). (**c**) Sustained release of tramadol (black squares) and of bupivacaine (red circles) from their metallic silver matrices composites. Full line: The fit to Weibull equation (equation (2), Table 4 (range of fit: 60 min).

The release profiles of the two entrapped acidic drugs and of the two basic drugs are shown in Fig. 4a and b, respectively. They are similar in their overall appearance, namely an initial faster region of release, followed by a slower one. Their compliance to release kinetic equations is, however, different. We begin with the two acidic NSAID drugs, ibuprofen and naproxen, compare the two composites to each other, and compare two formats, powder and compressed discs. Of several options of kinetic fits, the best fit was found with a model of two parallel first order release profiles with a slower (s) component and a faster (f) component (equation 2):

2 $${\rm{m}}({\rm{t}})={{\rm{m}}}_{{\rm{b}}}+{{\rm{m}}}_{{\rm{f}}}(1-\exp (-\frac{{\rm{t}}}{{{\rm{\tau }}}_{{\rm{f}}}}))+{{\rm{m}}}_{{\rm{s}}}(1-\exp (-\frac{{\rm{t}}}{{{\rm{\tau }}}_{{\rm{s}}}}))$$

where m_b_ is the fraction population of the drug that is released at the initial extraction (the “burst effect”), m_f_ and m_s_ are the fractions of the two main populations, and *τ*_f_ and *τ*_s_ are the characteristic time constants of the processes (namely 1/k_f_ and 1/k_s_, respectively, where k_f_ and k_s_ are the first-order process constants). Table 2 collects the parameters of the release profiles of the entrapped NSAIDs.

**Table 2:** The kinetic parameters of the release of ibuprofen and naproxen.

	ibu@Ag powder	ibu@Ag disk	nap@Ag powder	nap@Ag disk
m_b_ (%)	1.0 ± 0.1	2.0 ± 0.3	0 ± 0.5	0.15 ± 0.09
m_f_ (%)	50 ± 1	21 ± 3	58 ± 1	5 ± 1
m_s_ (%)	44 ± 1	79 ± 3	41 ± 1	75 ± 3
*τ*_f_ (t)	5.7 ± 0.2 min	0.15 ± 0.03 days	7.3 ± 0.3 min	0.17 ± 0.05 days
k_f_ (%/t)	0.18 ± 0.01 min	7 ± 1 days	0.14 ± 0.01 min	6 ± 2 days
*τ*_s_ (t)	220 ± 10 min	4.6 ± 0.5 days	240 ± 30 min	6.1 ± 0.6 days
k_s_ (%/t)	0.0045 ± 0.0002 min	0.22 ± 0.02 days	0.0042 ± 0.0005 min	0.16 ± 0.02 days
R^2^	0.999	0.998	0.999	0.998

As seen in Fig. 4a,b, the fit to the model of equation 2 is excellent. Indeed, this translates to very high correlation coefficients, R^2^ – see Table 2. It is also seen that m_b_ is very small in all cases – around 0–2% - indicating apparently some residual drug molecules that remained after the clean-washing of the composite; these molecules can be regarded as classically adsorbed, in contradistinction with the entrapped molecules that are sustained released - m_f_ + m_s_ (close to 100%). The rapidly released component is responsible for the release of  around 50% for ibu@Ag and somewhat higher, around 58%, for nap@Ag, with characteristic time constants of 5.7 and 7.3 minutes, and with process constants of 0.18% and 0.14% released/min, respectively. Compressing into discs, changes the ratio between the slow and fast populations, coincidentally into similar numbers – now the slower-released fractions are 79% and 75% for ibu@Ag and nap@Ag, respectively. This change of populations is explained by narrowing of the pores and the cage entrances due to the application of pressure. The characteristic time constants increase dramatically to 6–7 days, with process constants of 0.15 – 0.17%-released/day. It is shown therefore that pressure can be used as a control parameter for achieving release time scales from minutes to few weeks, and as a control parameter for achieving the desired ratio between the fast and slow populations. It is relevant to recall at this stage that drug release profiles which have a first fast, high concentration phase, followed by a prolonged slow release mode, is a desirable profile in many pharmaceutical formulations^31^.

The release kinetics of the two amine drugs from tra@Ag and bup@Ag (Fig. 4c) are also characterized by a fast mode and by a slow mode, but the best fit in this case is to the Weibull equation (equation 3), which already proved to be relevant for such metallic systems^23, 32^.

3 $${\rm{m}}({\rm{t}})={{\rm{m}}}_{\infty }\cdot (1-\exp ({(-\frac{{\rm{t}}}{{{\rm{T}}}_{{\rm{c}}}})}^{{\rm{b}}}))$$

Here m(t) is the dopant fraction that is released from the composite at time t, m_∞_ is the amount of the drug that is expected to be released at infinite time, T_c_ is a typical time constant of the system, which is defined as the time required for 63.2% of the entrapped dopant to be extracted (the condition t/T_c_ = 1 in equation 3), and b is a curve-shape parameter with a value range between 0 to 1. If b = 1, the equation is reduced to a simple first order equation with a homogeneous population of released molecules (see the components of equation 1), and the smaller–than-1 the parameter b is, the more is the profile more parabolic and with a higher initial slope. Thus (see Table 3) while the shape parameter of bup@Ag is 0.47, the shape parameter of tra@Ag is 0.29. Therefore we propose that bup@Ag is more homogenous compared to tra@Ag. Note also that while the extraction of tra is 100%, the extraction of bup is 73% (out of 100% of the TGA value), and where the slow release mode is longer. That slower extraction apparently reflects bupivacaine molecules that are entrapped within closed metallic aggregated cages – such residual un-extractable population of dopants entrapped in metals, have been observed before^25, 30^, and recently Pokroy *et al*. even found evidence for intercalation within the metallic crystal lattice in similar entrapment experiments of amino-acids in gold^33^. Finally, the two amine drugs did not provide good quality compressed discs (apparently this type of molecules has a lubricating effect on the metallic particles), which is therefore not reported.

**Table3:** The kinetic parameters of the release of tramadol and bupivacaine.

	tra@Ag	bup@Ag
m_∞_ (%)	100 ± 5	73 ± 4
T_c_ (min)	2.0 ± 0.2	15 ± 3
b	0.29 ± 0.02	0.47 ± 0.04
R^2^	0.97	0.98

Although the four entrapped drugs comply with two different (but related) models, comparison of all four is of interest, and this can be achieved by focusing on the initial release slopes (5 measured points after the first minute of the “burst effect”). As can be seen in Table 4, the initial release rates are of the same order of magnitude. This indicates that the main effect is due to the entrapping procedure itself and to the resulting caging of the drug molecules within the tightly held aggregates of the silver nanocrystals. The variability that is seen is then due to the different molecular moieties that interact with the walls of the silver cages. The slowing effect of the compression into discs is also seen in the table, and as mentioned above, can be used as a control-parameter of this property.

**Table 4:** Comparison of the initial release slopes of the four entrapped drugs.

	initial release rate %/min	initial release rate (mmol/min) × 10^−3^	R^2^
ibu@Ag	6.5	4.6	0.99
ibu@Ag disk	0.05	0.03	0.88
nap@Ag	6.9	4.8	0.99
nap@Ag disk	0.02	0.01	0.91
tra@Ag	7.5	3.7	0.96
bup@Ag	2.8	1.1	0.91

### The bacterial inhibition-growth activity

**Figure 5:**
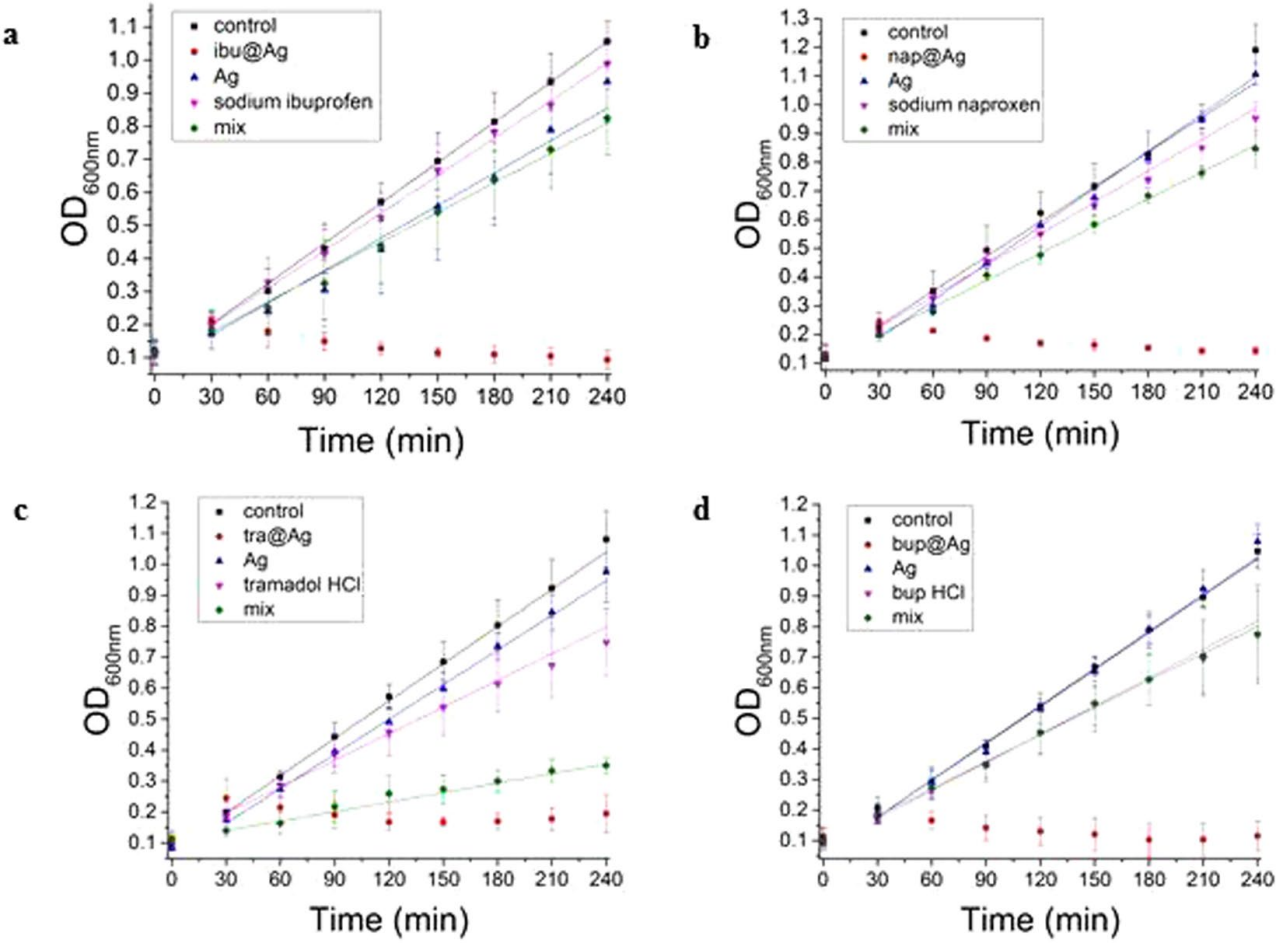
Bacterial (*P. aeruginosa* ﻿PAO1) growth as a function of time: (**a**) ibu@Ag, (**b**) nap@Ag, (**c**) tra@Ag, (**d**) bup@Ag. Total inhibition by the drug-silver composites: red dots at the bottom. This total inhibition is compared to several blanks, all at concentrations and amounts that follow the composites: Control (uninterrupted growth) – black; metallic silver prepared by the same method but without the dopant – blue triangles; the pure drug – down-pointing pink triangles; solution mixture of metallic Ag and the dissolved drug – green diamond. Zero point: before addition of the agents. 0.1 OD unit = 10^8^ CFU/ml.

We report now the results of *in vitro* bacterial growth inhibition studies of the new composites. As indicated in the introduction, a potential longer term applicative goal could be the control of both infection and pain in wound treatment, and therefore the opportunistic pathogenic *Pseudomonas aeruginosa*, a known wound contaminating bacterium was selected as a model to study of the inhibition of growth^34, 35^. We show that the entrapment of the analgesics in silver provides powerful growth inhibition agents, which by far exceed the activity of the two separate components, namely of pure metallic silver, and of the analgesic drugs. Except for tramadol, weak antibacterial activity has been reported for ibuprofen^36, 37^, naproxen^38^ and bupivacaine^39^; thus, a highly synergistic effect between the two components of the hybrid material – not seen in simple mixtures – is a key observation of this study; here are the details: We first focus on ibu@Ag. the results for which appear in Fig. 5a and in Table 5: The lower red dots in the figure indicate that total growth inhibition is achievable with this composite at the MIC concentration shown in Table 5 (The zero point in the figure is before adding the tested materials). On the other extreme of the figure we see the control, namely the uninhibited growth rate of the bacterial population. In between these two extremes we see several important blanks: First, just below the control are the inhibition dots of ibuprofen (same amount as in the composite), and it is seen that this anti-inflammatory drug does show some very weak growth-inhibition activity, which indeed was reported by Al-Janabi *et al*.^36^. Metallic silver (same amount as in the composite and prepared by the same procedure), as expected, shows better inhibition activity then the drug, but remarkably it is still by far less active than ibu@Ag. Even a simple mixture of metallic silver and the drug (green diamonds) shows no sign of the very strong synergism of the composite (in fact, slight inhibition of silver by the drug – probably due to adsorptive blocking), is seen. Quantitative values to this description are presented in Table 5, first row: While growth is totally inhibited for ibu@Ag (zero), compared to the control rate of 4.1(CFU ×10^6^)/(ml × min), silver alone and the drug alone reduce that rate to 3.3 and 3.8(CFU ×10^6^)/(ml × min), respectively, and slightly more – 3.0(CFU ×10^6^)/(ml × min) – for a simple mixture of the two; relative values are given in the Table as well (in italics).

**Table 5:** The biocidal activity of the four composites and comparison with blanks*.

	Ag: analgesic ratio by weight	MIC (mg/mL)	Relative MIC value	Bacterial growth rate				
				Drug@Ag	Control	Pure silver	Pure drug	Pure drug + Ag mixture
ibu@Ag	85.5: 14.5	17	0.12	0	**4.1**	**3.3**	**3.8**	**3.0**
				*1*	[0.999]	[0.969]	[0.999]	[0.996]
					*0*	*0*.*2*	*0*.*1*	*0*.*3*
nap@Ag	83.9: 16.1	5	0.4	0	**4.0**	**4.3**	**3.6**	**3.1**
				*1*	[0.998]	[0.998]	[0.999]	[0.999]
					*0*	*0*	*0*.*1*	*0*.*2*
tra@Ag	87.0: 13.0	50	0.04	0	**4.0**	**3.7**	**2.9**	**1.0**
				*1*	[0.998]	[0.997]	[0.989]	[0.997]
					*0*	*0*.*1*	*0*.*3*	*0*.*8*
bup@Ag	88.6: 11.4	2	1	0	**4.0**	**4.1**	**3.1**	**3.0**
				*1*	[0.996]	[0.993]	[0.999]	[0.998]
					*0*	*0*	*0*.*2*	*0*.*3*

^*^The fifth to the ninth columns read as follows: Within each box, first entry in bold - bacterial growth rate (CFU ×10^6^)/(ml × min), with an error range of approximately ± 0.1 (Fig. 5); second entry – the correlation coefficient, [R^2^], of the straight line fits (Fig. 5); third entry – the relative efficiency of inhibition compared to the composite.

Similar observations were made with the other three drug-composites (Fig. 5, Table 5), and we highlight the following: All four composites are very bioactive in inhibiting the bacterial growth. The most active composite is bup@Ag (the lowest MIC value – Table 5), while the least active is tra@Ag. The highest activity of bup@Ag is a reflection of the fact that the pure drug is the most active, but, as we shall see in the next section, not only for that reason. Interestingly, tra@Ag is the only case where the activity of the tra + Ag mixture approached that of the composite, probably because of the much higher amounts needed in this case.

As mentioned above, the composites affect the pH values during the MIC tests. For instance, the composite ibu@Ag induces slight acidity of pH 6.40. Pure ibuprofen acid had a slightly stronger effect on the growth compared to sodium ibuprofen, with an inhibition efficiency of 0.2 (see Fig. S6, Supplementary material). The basic environment for tra@Ag - pH = 7.55 - was created artificially, and the growth was shown to be unaffected. The durability of PAO1 to those pH ranges is supported in the literature^40^.

We focus now our attention on the release of Ag^+^, which is the well-known origin of the antibacterial action of silver^41–43^. Our hypothesis was that the high activity of the composites might originate, at least in part, from enhanced release of Ag^+^ due to the doping. Indeed, ICP-MS analyses of the Ag^+^ concentration values - Table 6 – suggest the hypothesis to be correct. It is seen (second row) that while pure silver prepared under similar conditions to that of the composites has an equilibrium concentration of around 17 ± 4 ppb/mg, higher values are observed for the composites: It is much higher for the most efficient bup@Ag - 1237 ppb/mg – and slightly higher for the least effective tra@Ag. Much higher concentrations are also observed for the two NSAIDs composites, and again we see that the more effective nap@Ag, releases 5 times more Ag^+^ compared to ibu@Ag. Why do the dopants promote the release of Ag^+^ from the metal? We have already observed this phenomenon in earlier studies of doped silver and suggested there that the adsorptive interactions of the molecule with the silver atoms at the interface of the metallic nanocrystal, weakens the crystal lattice bonding of those atoms with the rest of the crystal lattice^24^. In the case of the NSAIDs, it is mainly the carboxylate moiety that accelerates the detachment of the silver cation, and it is the tertiary amine moiety in bupivacaine which does it. Tramadol lacks such strong moieties, and therefore its effect is smaller. We recall that it has been suggested that the electrons which remain behind on the metallic silver, are captured by the dissolved oxygen, resulting in oxidized species such as H_2_O_2_^44^. We now have a plausible explanation for why bup@Ag is the strongest agent: It is a synergistic combination of the most active pure organic component, with the strongest promotion of Ag^+^ release. Furthermore, as can be seen in Table 5, 7th–9th columns, the mixture of tramadol or naproxen with silver inhibits the growth significantly better than expected, i.e., the relative efficiency of inhibition is higher than the sum of the pure dopant plus pure silver inhibitions. Following Kim *et al*.^45^ and Morones-Ramirez *et al*.^46^, we also suggest that another possible source of the synergism is that silver enhances the penetration of the drugs into the bacteria, amplifying the growth inhibition activity (and perhaps also the analgesic activity).

**Table 6:** Comparative Ag^+^ concentrations.

	ibu@Ag	silver	nap@Ag	silver	tra@Ag	silver	bup@Ag	silver
Ag^+^ (ppb)	3455	961	4817	218	2270	1812	6680	116
Ag^+^ ppb/mg	75.3	20.9	382.3	17.1	17.4	13.4	1237	21.5
Ratio of Ag^+^ released from composite/pure silver	3.6		22.1		1.3		57.6	
Relative Ag^+^ release-efficiency compared to bup@Ag	0.06	0.02	0.31	0.01	0.01	0.01	1.0	0.02

### Conclusions and outlook

We have proven in this report the concept that a metal can act as a 3D matrix for sustained release of drugs. Four drugs were used, and their detailed kinetics of release from silver were shown and analyzed. These observations add an important new family to the existing release matrices. Metals offer a unique set of properties, not found in the currently used matrices, and therefore a variety of potential new applicative directions are opened. One such potential application which led us to selection of silver doped with analgesics for the study of a metal as a releasing matrix, is wound treatment. Wounds provide an excellent hosting environment for benign as well as pathogenic bacteria whose presence not only delay the wound healing process, but can also lead to sepsis and death^15, 47–50^. About 60% of patients with chronic wounds are suffering from pain, which has major effects on their well-being and on the healing process^51, 52^. Furthermore, wound healing can be very costly and can take several months^53^. All of these factors call for new concepts in wound treatment, and the control of the microbial burden of the wound environment combined with potential pain relief activity, may prove to be such a new direction. In this context we mention examples of topical wounds preparations which are mixtures, such as morphine^54^ or bupivacaine with sulfadiazine^55^, or ibuprofen mixed with a foam of silver^56^. We also recall that topical treatment of analgesics with sustained release profiles is known for its benefits such as effective lower dose, direct access to the target site, long term efficacy, lower toxicity, and better patient compliance and convenience^7–9, 57^.

## Electronic supplementary material

Supplementary information

## References

[1] Cho K (2008). Therapeutic Nanoparticles for Drug Delivery in Cancer. Clin. Cancer Res..

[2] Iane BV (2012). Cytotoxicity and slow release of the anti-cancer drug doxorubicin from ZIF-. RSC Adv..

[3] Suvakanta D, Padala N, Murthy, Lilakanta N, Prasant C (2010). Kinetic Modeling On Drug Release from Controlled drug delivery Systems. Search Results Acta Pol. Pharm..

[4] Shaikh HK, Kshirsagar RV, Patil SG (2015). Mathematical Models for Drug Release Chracterization: A Review. WORLD J. Pharm. Pharm. Sci..

[5] Zalte HD, Saudagar RB (2013). Rewiew on sustained release matrix tablet. Int. J. Pharm. Biol. Sci..

[6] Gupta M, Brijesh RA (2012). Review on: Sustained Release Technology. Int. J. Ther. Appl..

[7] Jorge LL, Feres CC, Teles VE (2011). Topical preparations for pain relief: efficacy and patient adherence. J. Pain Res.

[8] Price P (2007). Why combine a foam dressing with ibuprofen for wound pain and moist wound healing?. Int. Wound J..

[9] Jørgensen B, Friis GJ, Gottrup F (2006). Pain and quality of life for patients with venous leg ulcers: Proof of concept of the efficacy of Biatain®-Ibu, a new pain reducing wound dressing. Wound Repair Regen..

[10] Martens M (1997). Efficacy and tolerability of a topical NSAID patch (local action transcutaneous flurbiprofen) and oral diclofenac in the treatment of soft-tissue rheumatism. Clin. Rheumatol..

[11] Baxter R, Bramlett K, Onel E, Daniels S (2013). Impact of Local Administration of Liposome Bupivacaine for Postsurgical Analgesia on Wound Healing: A Review of Data From Ten Prospective, Controlled Clinical Studies. Clin. Ther..

[12] Mattia C, Coluzzi F (2010). Tramadol: a wonder drug for the treatment of chronic pain?. Int. J. Clin. Rheumatol..

[13] Horcajada P (2006). Controlled release of Ibuprofen from dealuminated faujasites. Solid State Sci..

[14] Li Z (2014). Controlled release of liposome-encapsulated Naproxen from core-sheath electrospun nanofibers. Carbohydr. Polym..

[15] Shemesh M, Zilberman M (2014). Structure-property effects of novel bioresorbable hybrid structures with controlled release of analgesic drugs for wound healing applications. Acta Biomater..

[16] Liaw J, Lin Y (2000). Evaluation of poly(ethylene oxide)–poly(propylene oxide)– poly(ethylene oxide) (PEO–PPO–PEO) gels as a release vehicle for percutaneous fentanyl. Esevier.

[17] Barillo DJ, Marx DE (2014). ScienceDirect Silver in medicine: A brief history BC 335 to. Burns.

[18] Behar-Levy H, Avnir D (2002). Entrapment of Organic Molecules within Metals: Dyes in Silver. Chem. Mater..

[19] Behar-Levy H, Avnir D (2005). Silver Doped with Acidic/Basic Polymers: Novel, Reactive Metallic Composites. Adv. Funct. Mater..

[20] Ben-Knaz R, Avnir D (2009). Bioactive enzyme-metal composites: the entrapment of acid phosphatase within gold and silver. Biomaterials.

[21] Yosef I, Abu-Reziq R, Avnir D (2008). Entrapment of an organometallic complex within a metal: a concept for heterogeneous catalysis. J. Am. Chem. Soc..

[22] Avnir D (2014). Molecularly doped metals. Acc. Chem. Res..

[23] Ben-Knaz R, Pedahzur R, Avnir D (2010). A Concept in Bactericidal Materials: The Entrapment of Chlorhexidine within Silver. Adv. Funct. Mater..

[24] Ben-Knaz R, Pedahzur R, Avnir D (2013). Bioactive doped metals: high synergism in the bactericidal activity of chlorhexidine@silver towards wound pathogenic bacteria. RSC Adv..

[25] Ben-Knaz Wakshlak R, Pedahzur R, Menagen B, Avnir D (2016). An antibacterial copper composite more bioactive than metallic silver. J. Mater. Chem. B.

[26] Parsons D, Bowler P (2005). Silver antimicrobial dressings in wound management: a comparison of antibacterial, physical, and chemical characteristics. Wounds A Compend. Clin. Res. Pract..

[27] Yosef I, Avnir D (2006). Metal - Organic Composites: The Heterogeneous Organic Doping of the Coin Metals s Copper, Silver, and Gold. Chem. Mater..

[28] Salmerón JF (2014). Properties and printability of inkjet and screen-printed silver patterns for RFID antennas. J. Electron. Mater..

[29] Little, S. A., Begou, T., Collins, R. W. & Marsillac, S. Optical detection of melting point depression for silver nanoparticles via *in situ* real time spectroscopic ellipsometry. *Appl*. *Phys*. *Lett*. **100** (2012).

[30] Ben-Efraim Y, Avnir D (2012). Entrapment of organic molecules within binary metal alloys. J. Mater. Chem..

[31] Natarajan JV, Nugraha C, Ng XW, Venkatraman S (2014). Sustained-release from nanocarriers: A review. J. Control. Release.

[32] Sinai O, Avnir D (2009). Electrolytical entrapment of organic molecules within metals. J. Phys. Chem. B.

[33] Chen L, Polishchuk I, Weber E, Fitch AN, Pokroy B (2016). Hybrid Gold Single Crystals Incorporating Amino Acids. Cryst. Growth Des..

[34] Lyczak JB, Cannon CL, Pier GB (2000). Establishment of Pseudomonas aeruginosa infection: lessons from a versatile opportunist. Microbes Inferct..

[35] Cross A (1983). Nosocomial infections due to Pseudomonas aeruginosa: review of recent trends. Rev. Infect. Dis..

[36] Al-Janabi AAHS (2010). *In vitro* antibacterial activity of Ibuprofen and acetaminophen. J. Glob. Infect. Dis..

[37] Pin-Vaz C (2000). Antifungal activity of ibuprofen alone and in combination with fuconazole against Candida species. J. med. Microbiol.

[38] Dastidar SG, Ganguly K, Chaudhuri K, Chakrabarty AN (2000). The anti-bacterial action of diclofenac shown by inhibition of DNA synthesis. Int. J. Antimicrob. Agents.

[39] Johnson SM, Saint John BE, Dine AP (2008). Local anesthetics as antimicrobial agents: a review. Surg. Infect. (Larchmt)..

[40] Tsuji A, Kaneko Y, Takahashi K, Ogawa M, Goto S (1982). The effects of temperature and pH on the growth of eight enteric and nine glucose non-fermenting species of gram-negative rods. Microbiol. Immunol..

[41] Ovington LG (2004). The Truth about Silver. Ostomy Wound Manag..

[42] Russell AD, Hugo WB (1994). Antimicrobial Activity and Action of Silver. Prog. Med. Chem..

[43] Jung WK (2008). Antibacterial activity and mechanism of action of the silver ion in Staphylococcus aureus and Escherichia coli. Appl. Environ. Microbiol..

[44] Batchelor-mcauley C, Tschulik K, Neumann CCM (2014). Why are Silver Nanoparticles More Toxic Than Bulk Silver? Towards Understanding the Dissolution and Toxicity of Silver Nanoparticles. Int. J. Electrochem. Sci. Sci..

[45] Kim J, Pitts B, Stewart PS, Camper A, Yoon J (2008). Comparison of the antimicrobial effects of chlorine, silver ion, and tobramycin on biofilm. Antimicrob. Agents Chemother..

[46] Morones-Ramirez JR, Winkler Ja, Spina CS, Collins JJ (2013). Silver enhances antibiotic activity against gram-negative bacteria. Sci. Transl. Med..

[47] Orsted HL, Keast D, Forest L, Françoise M (2011). Basic Principles of Wound Healing. Wound Care Canada.

[48] Guo S, Dipietro La (2010). Factors affecting wound healing. J. Dent. Res..

[49] Sciences, B. & Building, J. A. Wound Healing Dressings and Drug Delivery Systems: A Review. **97**, 2892–2923 (2008). 10.1002/jps.21210 17963217

[50] Pruitt Ba, McManus aT, Kim SH, Goodwin CW (1998). Burn wound infections: current status. World J. Surg..

[51] Hoffman D (1997). Pain in venous leg ulcers. Journal of wound care.

[52] Solowiej K, Mason V, Upton D (2010). Psychological stress and pain in wound care, part 3: management. J. Wound Care.

[53] Dowsett, C. & Dowsett, C. Breaking the cycle of hard-to-heal wounds: balancing cost and care. *Wounds Int*. **6** (2015).

[54] Long TD (2001). Morphine-infused silver sulfadiazine (MISS) cream for burn analgesia: A pilot study. J. Burn Care Rehabil..

[55] Kleinbeck KR, Bader RA, Kao WJ (2013). Concurrenht *In Vitro* Release of silver sulfadiazine and bupivacaine from semi interpenetration networks for wound management. J. Burn Care Res..

[56] Jørgensen B (2008). Combined use of an ibuprofen-releasing foam dressing and silver dressing on infected leg ulcers. Journal of Wound Care.

[57] Flanagan, M., Vogensen, H. & Haase, H. Case series investigating the experience of pain in patients with chronic venous leg ulcers treated with a foam dressing releasing ibuprofen. *world wide wound* **1** (2006).

[58] The international center for diffraction data., http://www.icdd.com/products/pdf4.htm.

