# Supplementary material for: Sustained release from a metal - Analgesics entrapped within biocidal silver
Source: Sci Rep. 2017 Jun 23;7:4161. doi: 10.1038/s41598-017-03195-w (PMC5482836; doi:10.1038/s41598-017-03195-w)
Supplement: Supplementary file 1 — Supplementary information [file 41598_2017_3195_MOESM1_ESM.pdf]

Sustained release from a metal - Analgesics entrapped within biocidal silver

Barak Menagen[(a)], Rami Pedahzur[(b)] and David Avnir*[(a)]

[(a)] Institute of Chemistry and the Center for Nanoscience and Nanotechnology, the Hebrew University of Jerusalem, Jerusalem, 9190402, Israel, and [(b)]Department of Environmental Health, Hadassah Academic College, Jerusalem, 91010, Israel

* david.avnir@mail.huji.ac.il

Supplementary Material

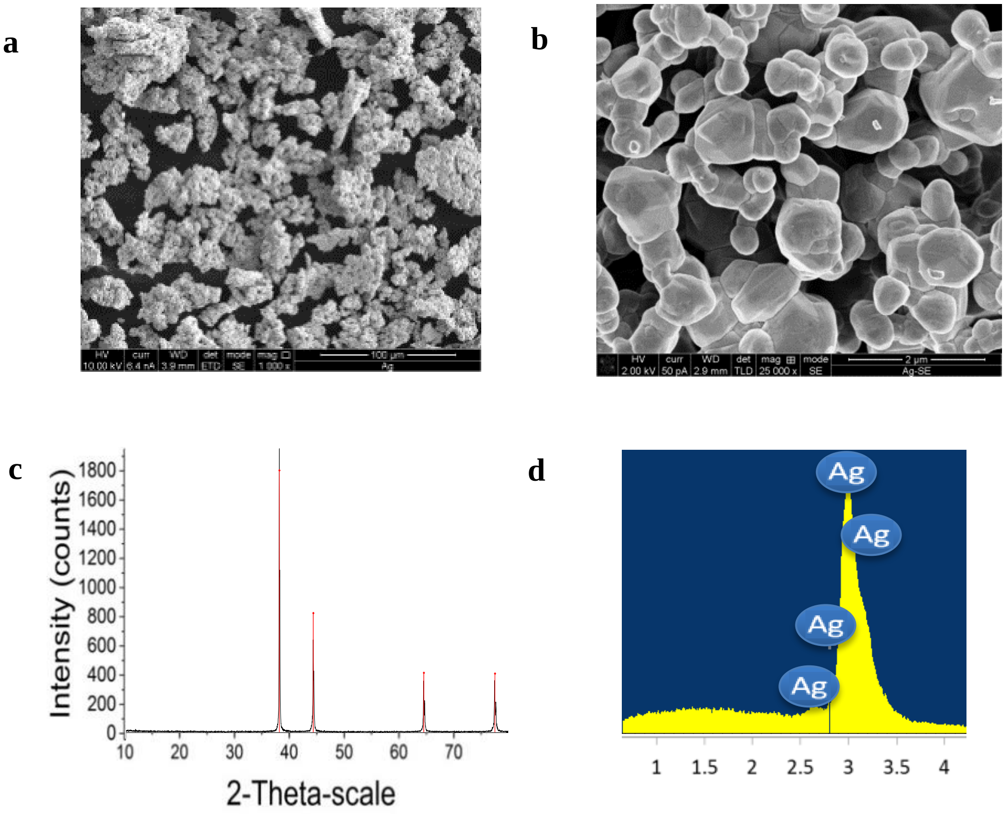

Figure S1. Pure silver prepared under the same conditions as in the doping experiments. SE-SEM: (a) bar - 100 µm, (b) bar - 2 µm. (c) XRD: black - the prepared Ag; red – literature data[1]. (d) EDAX – note that carbon and oxygen peaks are not seen.

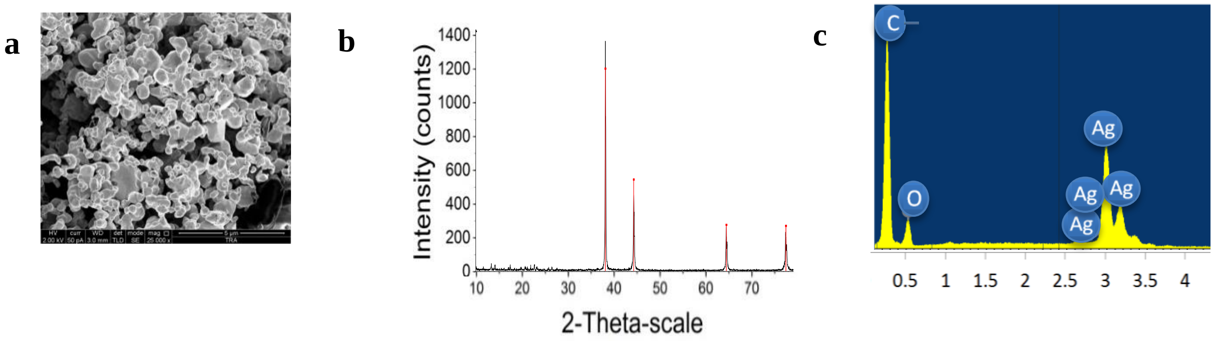

Figure S2. tra@Ag. (a) SE-SEM of tra@Ag; bar - 5 µm. (b) XRD: black – experimental, red – literature[1]. (c) EDAX – compare with Fig. S2 - O and C are clearly seen.

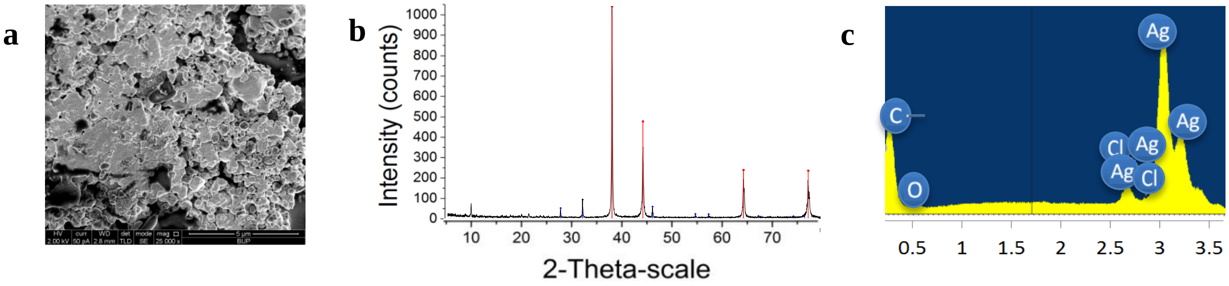

Figure S3. SE-SEM of bup@Ag: (a) bar - 5 µm. (b) XRD: black and red as in the previous figures. Blue – literature data silver chloride[1]. Silver: silver chloride ratio - 92.6 : 7.4. (c) EDAX.

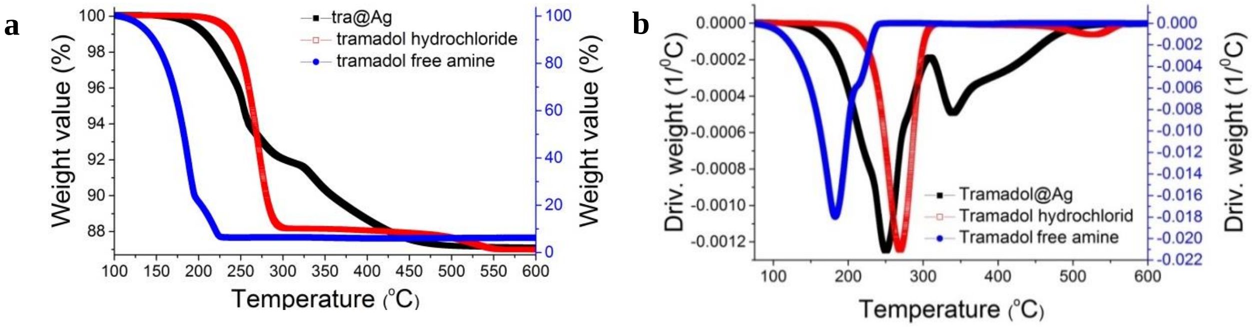

Figure S4. TGA profiles (left) and their derivatives (right) of tra@Ag, tramadol hydrochloride and the free tramadol amine. Left Y axis - the composite; right Y-axis the pure drugs.

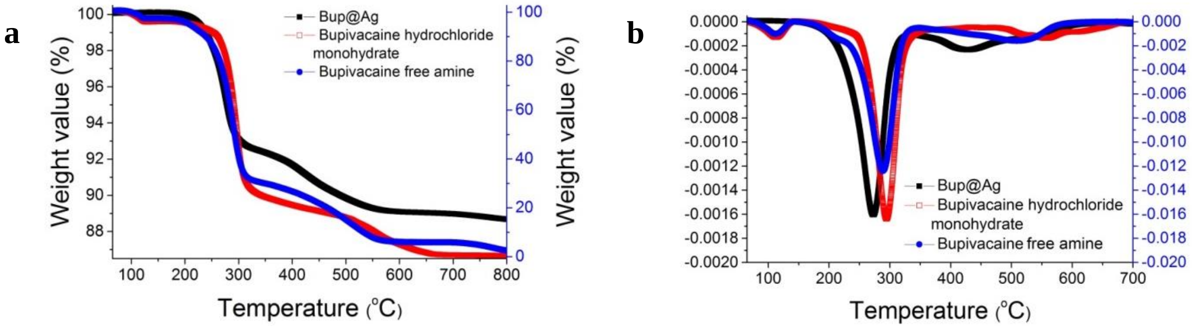

Figure S5. TGA profiles (left) and their derivatives (right) of bup@Ag, bupivacaine hydrochloride, and the free amine bupivacaine. Left Y axis - the composite; right Y-axis the pure drugs.

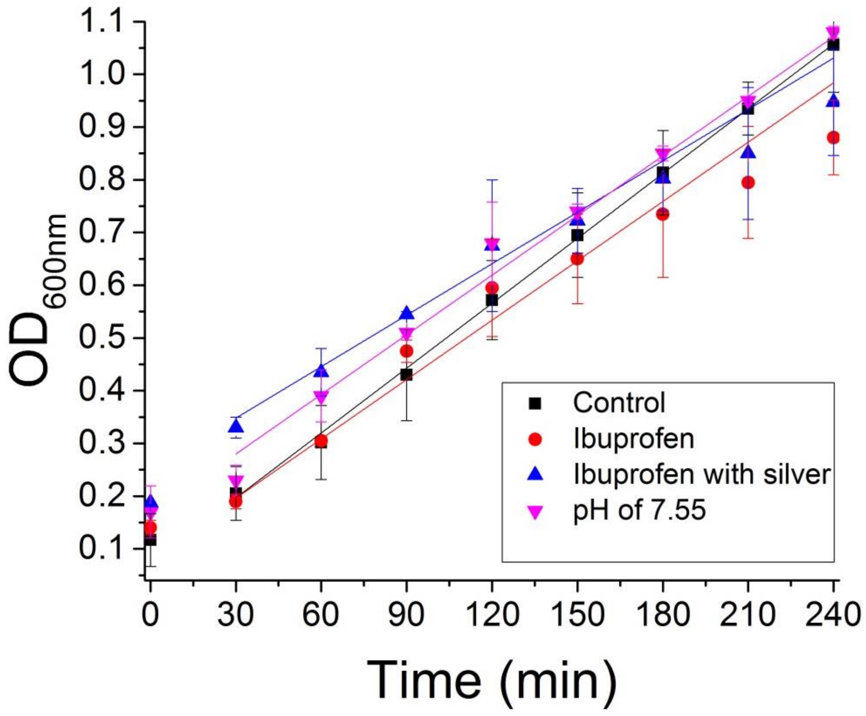

Figure S6. Some additional growth kinetics of PAO1, in the presence of ibuprofen (free acid), of ibuprofen (free acid) mixed with silver, at pH= 7.55 (compared to the control at pH = 7.00). The slopes are as follows (CFU $x10^6$)/(ml x min) - control: 4.1, ibuprofen: 3.2, ibprofen mixed with Ag: 2.9, pH of 7.55: 4.0.

References:

1. The international center for diffraction data., http://www.icdd.com/products/pdf4.htm
